# Antiviral Role of Surface Layer Protein A (SlpA) of *Lactobacillus acidophilus*

**DOI:** 10.3390/pathogens15010103

**Published:** 2026-01-19

**Authors:** Govindaraj Anumanthan, Ananta Prasad Arukha, Ayalew Mergia, Bikash Sahay

**Affiliations:** Department of Infectious Diseases and Immunology, UF College of Veterinary Medicine, Gainesville, FL 32608, USA; ganumanthan@ufl.edu (G.A.); ananta.arukha@ufl.edu (A.P.A.); mergiaa@ufl.edu (A.M.)

**Keywords:** norovirus, antiviral, microbiota

## Abstract

Norovirus is associated with vomiting and diarrhea and, in severe cases, can result in death. Currently, there is no effective treatment or vaccine for this virus. Bilateral interactions have been reported between gut microbiota and viral infection. Our laboratory has been studying the Surface layer protein A (SlpA) of a human isolate of *Lactobacillus acidophilus.* Previously, we reported that SlpA induces a variety of antiviral genes in human dendritic cells, suggesting it may prevent viral replication. SlpA binds to its cognate receptor SIGNR3-expressed on limited dendritic cells. To achieve a homogenous expression of the gene, we modified murine macrophage RAW 264.7 (RAW) cells by transducing SIGNR3-expressing lentivirus, resulting in RAWS cells. These cells and wild-type RAW cells were pretreated with SlpA for one hour and infected with 1 MOI of murine norovirus (MNV). We report that RAWS cells, when treated with SlpA, enhance the antiviral program to prevent viral replication, as determined by quantitative real-time PCR and viral titer. RNA isolated from MNV-infected cells revealed an elevation in two critical antiviral genes, *Iigp1* and *Ifit1*, in SlpA-treated RAWS cells, potentially preventing viral replication.

## 1. Introduction

Human Norovirus (HuNoV), previously known as Norwalk virus, is a highly contagious, non-enveloped, positive-sense single-stranded RNA virus classified within the *Caliciviridae* family [1]. It is the leading etiological agent of acute gastroenteritis across all age demographics in the United States [2]. Clinical manifestations include severe gastrointestinal inflammation characterized by vomiting, diarrhea, abdominal pain, fever, and dehydration, typically resolving within 24–48 h. However, in immunocompromised and vulnerable populations, Norovirus infection can result in fatal outcomes [3].

Transmission occurs primarily via the fecal–oral route, either through direct person-to-person contact or indirectly via contaminated food, water, or aerosolized vomitus. Its extreme infectivity—requiring fewer than 100 viral particles to initiate infection—facilitates rapid and widespread outbreaks [2]. Consequently, Norovirus is responsible for an estimated 56,000–71,000 hospitalizations, 400,000 emergency department visits, and 570–800 deaths annually in the United States, with a global burden of approximately 699 million cases and 219,000 deaths [4].

Despite its profound public health impact, vaccine development for Norovirus remains elusive. As an RNA virus, Norovirus relies on an error-prone RNA-dependent RNA polymerase, resulting in frequent point mutations and recombination events that drive its rapid evolution [5]. Compounding this challenge is the uncertainty surrounding the duration of protective immunity. While early studies suggested immunity may last from two months to two years [6], more recent modeling indicates a potential range of 4–8 years [7]. This high genetic variability, coupled with limited understanding of long-term immune protection, has significantly hindered the development of a broadly effective vaccine. Currently, supportive care through fluid and electrolyte replacement remains the only available treatment [8].

In light of these challenges, alternative strategies have emerged, including the use of Murine Norovirus (MNV) as a surrogate model. MNV shares key biochemical and genetic characteristics with HuNoV—such as size, morphology, and buoyant density—making it a valuable proxy for studying viral survival, inactivation, and host–pathogen interactions [9,10]. The cost-effectiveness and genetic tractability of murine models further enhance their utility in investigating Norovirus pathogenesis and immune responses.

*Lactobacillus acidophilus* is a part of the healthy human intestine. The probiotic nature of the bacterium has been attributed to its metabolites and surface proteins, which elicit a protective immune response against various inflammatory stimuli. One such bacterium, isolated from the human intestine, has been well characterized and marketed as a probiotic [11,12,13,14,15]. The surface-associated macromolecular structures on these probiotic bacteria contribute to their protective behavior [16,17]. Some of these surface molecules are important for the survival of the bacterium in the intestinal environment [18,19,20,21] and others act as effector molecules by triggering immunological signals [22,23,24]. Surface-protein characterization of *L. acidophilus* NCFM (North Carolina Food Microbiology) (NCBI taxonomic ID: txid272621) revealed that its surface is composed of multiple proteins. One such protein is the Surface Layer Protein A (SlpA), a 47 kDa protein present in other S-layer-forming lactobacilli, such as *L. helveticus*, *L. crispatus*, and *L. amylovorus*, with varying sequence identity. SlpA isolated from *L. acidophilus* is well studied. Initially, SlpA was identified as an anchoring protein required for bacterial attachment to intestinal epithelial cells. However, recent studies from our laboratory and others have shown the immunomodulatory properties of SlpA from *L. acidophilus* NCFM. We described the immunomodulatory properties of SlpA, based on its interaction with a C-type lectin receptor, SIGNR3, in mice [23]. SIGNR3, explicitly expressed on dendritic cells, and its engagement by SlpA modulates epithelial, myeloid, and T-cell functions, conferring protection in murine colitis models [24]. Our previous transcriptomic analysis revealed upregulation of antiviral gene signatures following SlpA recognition by human dendritic cells [25], suggesting a potential antiviral mechanism.

Based on these findings, we hypothesize that SlpA-SIGNR3 interaction may inhibit viral replication. This hypothesis was tested using genetically engineered RAW 264.7 cells, where it showed that SlpA interaction with its cognate receptor SIGNR3 leads to an antiviral status, potentially increasing antiviral genes, such as *Iigp1* and *Ifit1*. More studies are required to verify its applicability in the murine model of infection.

## 2. Materials and Methods

Virus: Murine Norovirus, CW3 strain was obtained from the Biodefense and Emerging Infections Research Resources Repository (BEI Resources; Product ID NR-50895). For viral propagation, RAW 264.7 cells (Cat. # TIB-7; ATCC, Manassas, VA, USA), a murine macrophage cell line, were infected at an MOI of 0.05, and the supernatant was collected after 48 h. The collected supernatants were stored in −80 °C and titrated using the RAW 264.7 murine macrophage cell line. RAW 264.7 cells were maintained in the laboratory in Dulbecco’s Modified Eagle’s Medium (DMEM), (Cat# 11965092, Gibco, Carlsbad, CA, USA) with 10% Fetal Bovine Serum (FBS, Cat# A5256701, Gibco, Carlsbad, CA, USA).

Generation of SIGNR3-expressing RAW 264.7 cells: Lentivirus expressing murine CD209d (SIGNR3) was purchased from Vector Builder Inc., (Chicago, IL, USA). The lentivirus has inbuilt green fluorescent protein expression, which was used for sorting the fluorescently isolated transduced cells using the SONY SH800S Cell Sorter (San Jose, CA, USA) available at the UF College of Veterinary Medicine. Briefly, a million RAW 264.7 cells were placed in a well of a six-well plate for four hours for their adherence to the surface. The lentiviral particles were thawed on ice, and 10 × 10^6^ viral particles were mixed with 8 μg/mL polybrene (supplied by the Vector Builder) in 1.5 mL of DMEM complete media (with 10% FBS). These cells were incubated at 37 °C with 5% CO_2_ for 48 h. These cells were washed with cold PBS and scraped off the surface using a cell scraper. The cells were washed twice with PBS and finally placed in DMEM media with 2% FBS. These cells were used for cell-sorting to collect GFP+ cells using a SONY SH800S. Un-transduced cells were used as a control to determine the GFP+ cells. A total of 50,000 cells were collected in this process, which were placed in the growth media for a week in a 12-well flat-bottom tissue-culture-treated cell culture plate (Cat#3513; Corning, NY, USA), and GFP expression in these cells was verified using fluorescent microscopy (KEYENCE BZ-X800 series fluorescence microscope, Itasca, IL, USA). These cells were designated as RAWS.

SlpA isolation: Surface layer protein A was isolated from the NCK2187 bacterial strain available in the laboratory using the 5M NaCl method, described elsewhere [24,26]. Briefly, the NCK2187 strain of *L. acidophilus*, which is devoid of SlpB, SlpX, and Lipoteichoic acid (LTA), leaving behind SlpA, was grown for 18 h in MRS media (Cat # DF0881-17-5; BD Biosciences, Sparks, MD, USA) under anaerobic conditions at 37 °C. The bacteria were collected by centrifuging at 5000× *g* for 10 min. The bacterial pellet was washed with cold PBS, resuspended in 5M sodium chloride (NaCl) and incubated at room temperature for 30 min with mild agitation. Afterwards, the bacterial pellet was collected and removed as before, and the supernatant was dialyzed against deionized water to remove NaCl. The precipitated protein after dialysis was collected by centrifugation at 23,000× *g* for 30 min at 4 °C. The isolated protein sample was resuspended in 1M NaCl and centrifuged at 23,000× *g* for 30 min at 4 °C. The pellet was collected and resuspended in deionized water. The concentration of the isolated protein was evaluated using Pierce BCA Protein Assay Kits (Thermo Fisher Scientific, Waltham, MA, USA; Cat 23227) as per the manufacturer’s instructions.

Cellular stimulation and MNV infection: RAW 264.7 cells or RAWS were seeded a day before the cellular stimulation. The next day, these cells were pre-stimulated for four hours with SlpA and then were exposed to 1 or 10 MOI of MNV. After one or two days of infection, either supernatants or the cellular contents were kept for virus evaluation by cellular titration to get the TCID_50_ or relative quantification using real-time PCR.

RNA Isolation and cDNA preparation: Total RNA was isolated from the cells using the Directzol RNA isolation kit (Cat # Z5227; Zymo Research, Irvin, CA, USA). The isolated RNAs were converted into cDNA for quantitative evaluation of transcripts using LunaScript RT SuperMix (Cat # M3010L; NEB biolabs, Ipswich, MA, USA) according to the manufacturer’s instructions. The concentration of isolated RNA was measured using Biodrop (Harvard Bioscience, Inc., Holliston, MA, USA).

Semi-quantitative Real-time PCR: 500 ng RNA of each sample was converted into 20 μL of cDNA. For the final 10 μL reaction, 0.2 μL of cDNA was mixed with 5 μL of SsoAdvanced Universal SYBR Green Supermix (Cat # 1725270; Bio-Rad Laboratories, Inc., Hercules, CA, USA) mixed with 10 pmol of both forward and reverse primers (Table 1). qPCR was performed on a MIC qPCR machine (Bio Molecular Systems, Coomera, QLD, Australia). All the qPCR reactions were run in triplicate with no-template controls (NTC), and mean cT values were used for all the calculations using 18S rRNA as an internal normalization control. Transcript levels for infected groups are presented as a fold change over their corresponding uninfected control group. Amplification conditions were 95 °C (3 min) and 40 cycles of 95 °C (15 s), 55 °C (40 s), and 72 °C (30 s).

## 3. Results

### 3.1. Construction of Chimeric RAW 264.7 Cells Expressing the Murine SIGNR3 Gene

**Figure 1:**
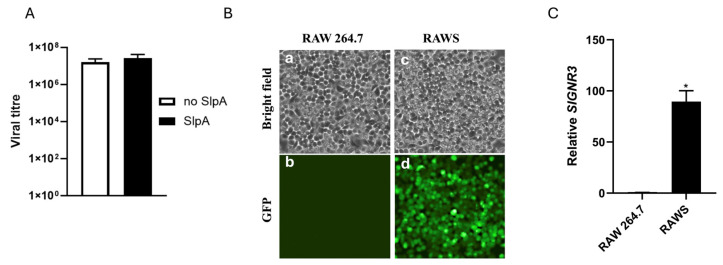
Generation of murine SIGNR3 stably expressing RAW 264.7 cells. (**A**) TCID50 assay performed on MNV-infected RAW 264.7 cells with or without SlpA. RAW 264.7 cells were pretreated with SlpA (10 ng/mL) for 4 h, then infected with MNV at an MOI of 1.0. Cells were incubated at 37 °C with 5% CO_2_ for 24 h. Viral titers were determined using the TCID50 assay. (**B**) RAW 264.7 cells were transduced with lentivirus expressing murine SIGNR3-GFP. After 48 h, cells were sorted for SIGNR3-GFP expression using a Sony SH800S cell sorter. The sorted cells were further cultured under normal growth conditions for further analysis. (a) Bright field image of RAW 264.7 cells, (b) Fluorescent image of RAW 264.7 cells, (c) Bright Field image of SIGNR3 expressing RAW264.7 cells (RAWS), and (d) Fluorescent image of RAWS cells. (**C**) Transcript analysis of SIGNR3 between RAW 264.7 and RAWS using 18sRNA as an internal control. * *p* < 0.05.

In a previous RNAseq analysis, we reported that SlpA induces several antiviral genes in human dendritic cells [25]. To determine whether SlpA affects viral replication, we sought a virus that replicates in myeloid cells. Murine Norovirus CW3 is a myeloid-tropic virus that replicates on the mouse macrophage cell line RAW 264.7 (RAW). SlpA interacts with SIGNR3 on dendritic cells to exert its function, which is absent in RAW cells. Consequently, SlpA treatment did not affect viral titers in cells that do not express SIGNR3 receptors (Figure 1A). To test whether SlpA has antiviral properties, we expressed SIGNR3 in the RAW 264.7 (RAWS) cell line via lentiviral transduction. The lentiviral particle also expressed Green Fluorescent Protein, which we used for cell sorting. Cells were transduced according to the manufacturer’s instructions. After 48 h post-transduction, transduced cells were sorted in a cell culture plate based on the GFP expression using the SH800 Sony cell sorter. Figure 1B shows the GFP expression as SIGNR3 expression in transduced cells, where untransduced cells show no GFP expression. Total RNA was isolated from RAW 264.7 and RAWS cells, and SIGNR3 transcript levels were compared between the two cell types, with 18S rRNA as an internal control. The data showed increased SIGNR3 transcript levels in RAWS compared with RAW 264.7 cells, and the two groups differed significantly by Student’s *t*-test with Welch’s correction (Figure 1C).

### 3.2. SlpA Treatment Prevents MNV Replication

**Figure 2:**
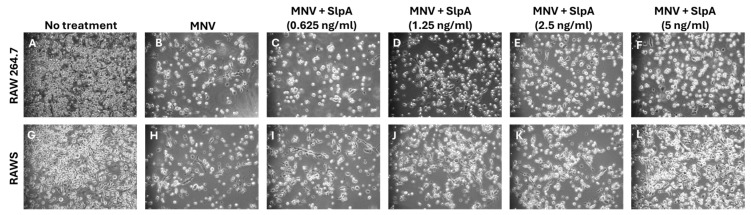
Optimization of SlpA concentration. Both RAW 264.7 and SINGR3-GFP expressing RAW 264.7 cells (RAWS) (1 × 10^5^ cells/well) were plated in 6-well plates. After 24 h, cells were treated with increasing concentrations of SlpA for four hours. MNV (MOI: 1) was treated in all wells except controls, as shown above. Cells without MNV and SlpA were considered as no-treatment controls. After 48 h of MNV treatment, cells were evaluated under a bright-field microscope for cytopathic effects of MNV. Concentration-dependent effects were observed, as shown above (**A**–**F**) for RAW 264.7 cells, (**G**–**L**) for SIGNR3-GFP-RAW 264.7 cells (RAWS).

To evaluate the antiviral role of SlpA, we pretreated RAW 264.7 and RAWS cells with increasing concentrations of SlpA (0.625 ng/mL to 5 ng/mL) diluted in cell culture media for 4 h before MNV infection at an MOI of 1. Forty-eight hours post-infection, fewer cells remained in the wells of RAW 264.7 cells, regardless of SlpA treatment. However, we found more live cells at the five ng/mL concentration compared to other concentrations of SlpA used in the experiment, suggesting a clear cytoprotective role of SlpA during viral infection (Figure 2). Three independent experiments were conducted to verify the results.

**Figure 3:**
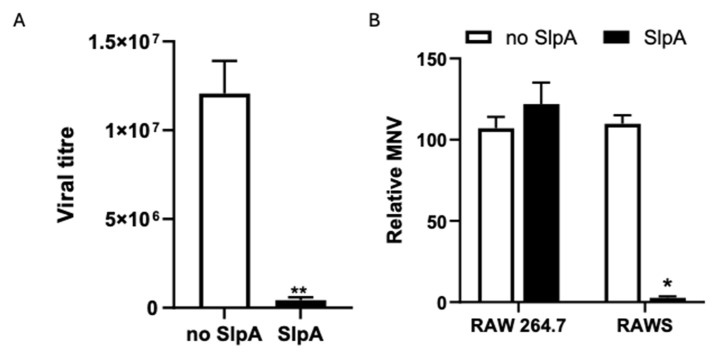
SlpA suppresses MNV replication in RAWS cells. RAW 264.7 and SIGNR3-GFP expressing RAW 264.7 (RAWS) cells were treated with or without SlpA (10 ng/mL) for 4 h before infection with MNV (MOI: 10). (**A**) Forty-eight-hour post-infection of RAWS cell-supernatants were collected for viral titre determination using TCID50 assay. (**B**) Twenty-four hours post-infection, total RNA was isolated from the samples. The cDNA converted from the isolated RNA was evaluated for the presence of viral genome using semi-quantitative real-time PCR. 18S rRNA was used as an endogenous control, and data were presented as the relative abundance of MNV genome compared with RAW 264.7 infected cells. Three independent replicates were performed. A statistically significant difference (*p* ≤ 0.05) was determined by the Mann–Whitney test, denoted by an asterisk. (N = 3; * *p* < 0.05); ** *p* < 0.01.

Later, we repeated this experiment with a 10 ng/mL concentration of isolated SlpA and an MOI of 10 for 24 h (i) to ensure homogenous interaction between cells and the virus, and (ii) to shorten the timeline of the experiment to isolate the RNA for transcriptomic analysis. The supernatants were collected to evaluate the viral load by the conventional TCID_50_ method [29]. SlpA-treated RAWS prevented viral replication by approximately 287-fold (viral titer mean of untreated RAWS cells was 1.21 × 10^7^, and viral titer mean of SlpA-treated RAWS cells was 4.25 × 10^5^) (Figure 3A). Total RNA was isolated to evaluate the presence of viral RNA in the samples. Semiquantitative real-time PCR was used to determine the relative quantity of viral RNA. RNA isolated from uninfected cells served as a control. To determine the viral RNA, the median MNV viral load in MNV-infected cells was set to 100% to estimate the relative MNV load in samples pre-treated with SlpA. The results indicate that in RAW 264.7 cells, the presence of viral RNA remains unchanged with or without SlpA stimulation (Figure 3B), suggesting that SlpA does not prevent viral replication in these cells. However, viral RNA levels were significantly lower in RAWS cells treated with SlpA compared to those that did not receive SlpA treatment (Figure 3B). These data represent the results of three independent experiments.

### 3.3. Presence of Signr3 and SlpA Treatment Enhances Antiviral Gene Signature

**Figure 4:**
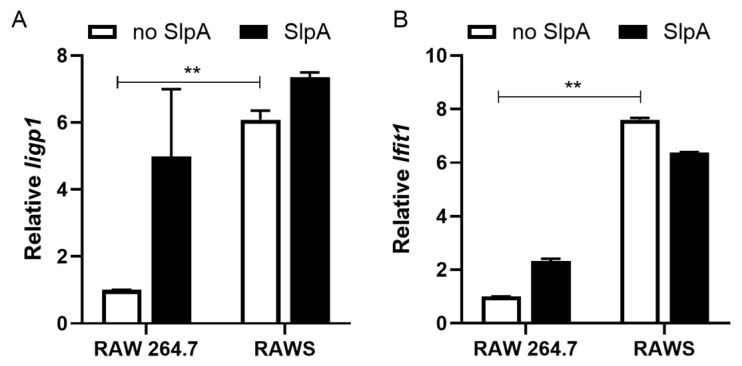
The presence of Signr3 and SlpA treatment enhances antiviral gene expression. RAW 264.7 and SIGNR3-GFP expressing RAW 264.7 (RAWS) cells were treated with or without SlpA (10 ng/mL) for 24 h. Total RNA was isolated from the samples. The cDNA converted from the isolated RNA was evaluated for the presence of (**A**) *Iigp1* and (**B**) *Ifit1*-specific transcripts using semi-quantitative real-time PCR. 18S rRNA was used as an endogenous control, and data were presented as the relative abundance of *Iigp1* and *Ifit1* transcripts compared with untreated RAW 264.7 no-SlpA-treated controls. N = 3; ** *p* < 0.01.

Previously, we reported a few antiviral genes elevated in human dendritic cells upon recognition of SlpA [25]. We evaluated several antiviral genes for their transcriptional changes. The *Iigp1* gene encodes an IFN-induced GTPase that is known to prevent norovirus replication [30]. Another antiviral gene, *Ifit1*, encodes an antiviral factor that promotes the antiviral state following cytoplasmic RNA sensing, thereby restricting norovirus replication [31]. When these transcripts of these two genes were evaluated in RAW 264.7 and RAWS cells in the presence and absence of SlpA, we found that the expression of these genes increased moderately in the presence of SlpA in RAW 264.7 cells. However, expression of these genes was significantly elevated in the RAWS, even in the absence of SlpA treatment (Figure 4A,B). The transcription of *Iigp1* further increased with SlpA-treatment, but the expression of *Ifit1* reduced moderately with the treatment (Figure 4A,B).

**Figure 5:**
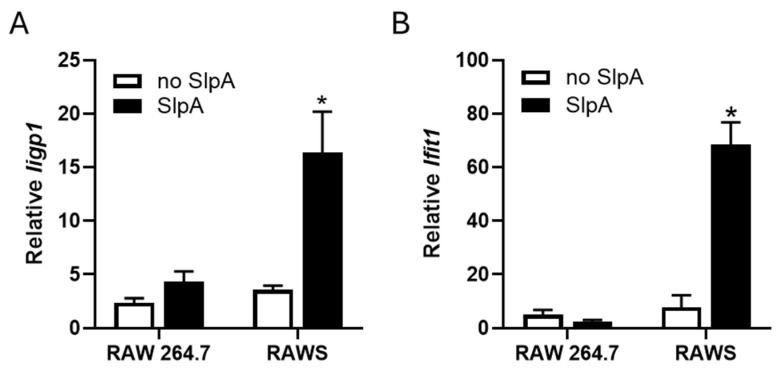
SlpA treatment enhances antiviral gene expression during MNV infection. RAW 264.7 and SIGNR3-GFP expressing RAW 264.7 (RAWS) cells were pretreated with or without SlpA (10 ng/mL) for 4 h before the infection with MNV (MOI: 1.0). Twenty-four hours post-infection, total RNA was isolated from the samples. The cDNA converted from the isolated RNA was evaluated for the presence of (**A**) *Iigp1* and (**B**) *Ifit1*-specific transcripts using semi-quantitative real-time PCR. 18S rRNA was used as an endogenous control, and data were presented as the relative abundance of *Iigp1* and *Ifit1* transcripts compared with their uninfected, no-SlpA-treated controls. N = 3; * *p* < 0.05.

A similar experiment was carried out in cells infected with 1 MOI of MNV for 24 h after pretreatment with SlpA for four hours. The semi-quantitative real-time PCR data showed that *Iigp1* transcription increased 16-fold in the infected RAWS cells pretreated with SlpA compared to untreated cells. In contrast, in other groups (RAW 264.7 infected with and without SlpA treatment and RAWS cells infected without SlpA treatment), transcription increased by 2–4-fold, depending on the group (Figure 5A).

Upon transcriptional analysis, we found that SlpA-treated MNV-infected RAWS cells showed a 68-fold increase in transcription compared to the uninfected, untreated cells. On the other hand, other groups showed a 2- to 7-fold increase in transcription across three independent experiments (Figure 5B).

## 4. Discussion

We and others have shown the immunoprotective role of the Surface layer protein A (SlpA) of *Lactobacillus acidophilus* [23,24,25]. We also showed that SlpA interacts with SIGNR3 expressed on dendritic cells to confer protection against inflammatory diseases, such as colitis [24,25]. RNAseq analysis using human DCs revealed that a group of antiviral genes was elevated in response to SlpA recognition. Based on these findings, we wondered whether SlpA recognition could prevent viral infections. SIGNR3 is expressed on a small subset of murine DCs, and due to the lack of any suitable antibodies, it is impossible to enrich the population for their evaluation. We genetically engineered a murine macrophage cell line expressing SIGNR3 using a lentiviral vector system. Similar methods have been used earlier, and due to the identical origin of macrophages and DCs, we anticipated that similar signaling components would be available for SlpA-interaction [32]. We chose to use Murine Norovirus for our study for two reasons: (i) a general focus of our laboratory in intestinal inflammation, and (ii) the ability of MNV to infect RAW 264.7 cells. Again, the use of CW3 strain was used because they are macrophage tropic [33].

Surface layer (S-layer) proteins are widely distributed among Lactobacillus species and represent the outermost component of the bacterial cell envelope [34]. Despite structural similarities, their primary sequences vary considerably, even among closely related strains. These proteins form a para-crystalline lattice that provides cell protection, mediates adhesion, and modulates host immune responses [35]. SlpA, a major S-layer protein in many Lactobacillus species, consists of two functional domains: a self-assembly domain that enables lattice formation on the cell surface and a cell wall-binding domain that anchors the protein to peptidoglycan or teichoic acids through non-covalent interactions, ensuring S-layer integrity [36]. Functionally, SlpA contributes to bacterial adherence, biofilm formation, and immunomodulation, which are critical for probiotic activity. Although S-layer proteins share physical and biochemical properties, they exhibit distinct immunological effects [37,38]. For example, SlpA from *Lactobacillus helveticus* and *L. acidophilus* both suppress NF-κB activity in human epithelial cells; however, SlpA from *L. helveticus* induces TNF production in human monocytic cells via TLR2 and does not alter IL-10 secretion [39], whereas SlpA from *L. acidophilus* promotes IL-10 production both in vitro and in vivo [23,24,25]. These findings underscore strain-specific immunomodulatory roles of S-layer proteins and their potential impact on host immune homeostasis.

Previously, we found an increase in antiviral genes in human DCs when treated with SlpA [25]. Thus, we chose to study two critical antiviral genes, *Iigp1* and *Ifit1* [31,40]. Both genes were found to be elevated in RAWS cells treated with SlpA, whereas in RAW 264.7 cells, their expression was limited and did not differ with SlpA treatment. *Iigp1* is responsible for innate immunity by trafficking pathogens intracellularly through its interaction with the Golgi apparatus [41], and *Ifit1* limits autoimmune inflammation within the cell. However, both genes belong to the interferon-induced gene family, known for their antiviral activity.

Upon the transcriptional analysis of *Ifit1* and *Iigp1*, a unique feature was revealed: without any SlpA stimulus, RAWS cells transcribe more of these antiviral genes compared to the parent RAW 264.7 cells (approximately 6-8-fold higher). This increase in these antiviral genes, in the absence of SlpA, suggests that SIGNR3 may recognize endogenous ligands that are not yet known to us but have been reported for other C-type lectins [42,43]. When SlpA was treated without infection, RAWS cells did not increase the transcription of these genes; however, during infection, transcription of the same genes increased several-fold. During viral infection, viral ligands may exert synergistic effects that enhance the transcription of these genes.

In future studies, we will focus on (i) cellular signaling triggered by the interaction between SlpA and SINGR3, (ii) testing the SlpA in a mouse model of infection, and (iii) developing a human in vitro system to validate these findings. Ongoing studies include global transcriptomic and proteomic changes upon SlpA-SIGNR3 interactions.

## 5. Conclusions

*Lactobacillus acidophilus* is a commensal bacterium living in the human gut. There are several ways the bacterium is known to exert its beneficial effects on the human gut. We demonstrate that SlpA pretreatment confers significant protection against murine norovirus infection in RAW 264.7 cells engineered to express SIGNR3 (RAWS). This protective effect is accompanied by robust induction of key antiviral immune genes, including *Iigp1* and *Ifit1*, suggesting activation of innate defense pathways. These findings not only underscore the immunomodulatory potential of SlpA but also lay the groundwork for advancing this strategy into in vivo murine models to evaluate its efficacy against gastrointestinal viral infections.

## 6. Patents

B.S. is the inventor of record on a patent held by the University of Florida and may be entitled to royalties from companies developing commercial products related to the research described in this paper. The corporate funders had no role in the design of the study, in the collection, analysis, or interpretation of data, in the writing of the manuscript, or in the decision to publish the results.

## Figures and Tables

**Table 1:** Primers.

Primers	Sequences	Reference
MNV_F	5′-ATGGTA/GGTCCCACGCCAC-3′	[27]
MNV_R	5′-TGCGCCATCACTCATCC-3′	
Iigp1_F	5′-GTAGTGTGCTCAATGTTGCTGTCAC-3′	This Study
Iigp1_R	5′-TACCTCCACCACCCCAGTTTTAGC-3′	
Ifit1_F	5′-TACAGGCTGGAGTGTGCTGAGA-3′	This Study
Ifit1_R	5′-CTCCACTTTCAGAGCCTTCGCA-3′	
18sRNA_F	5′-ATAGCGTATATTAAAGTTG-3′	[28]
18sRNA_R	5′-GTCCTATTCCATTATTCC-3′	
SIGNR3_F	5′-GGTCATTCCAGAGGATGAAGAG-3′	This Study
SIGNR3-R	5′-TCTTTGGGACTTGGAGAAGAAG-3′	

## Data Availability

The original contributions presented in this study are included in the article. Further inquiries can be directed to the corresponding author.

## Abbreviations

The following abbreviations are used in this manuscript:

RAW	RAW 264.7 cells
RAWS	RAW 264.7 cells expressing murine SIGNR3 cells
SlpA	Surface Layer Protein A
